# In Vivo Characterization of Avian Influenza A (H5N1) and (H7N9) Viruses Isolated from Canadian Travelers

**DOI:** 10.3390/v11020193

**Published:** 2019-02-23

**Authors:** Yao Lu, Shelby Landreth, Amit Gaba, Magda Hlasny, Guanqun Liu, Yanyun Huang, Yan Zhou

**Affiliations:** 1 Vaccine and Infectious Disease Organization - International Vaccine Centre (VIDO-InterVac), University of Saskatchewan, Saskatoon, SK S7N 5E3, Canada; yao.lu@usask.ca (Y.L.); shelby.landreth@usask.ca (S.L.); amg340@mail.usask.ca (A.G.); mth280@mail.usask.ca (M.H.); guanqun.liu@usask.ca (G.L.); 2 Department of Veterinary Microbiology, Western College of Veterinary Medicine, University of Saskatchewan, Saskatoon, SK S7N 5B4, Canada; 3 Vaccinology & Immunotherapeutics program, School of Public Health, University of Saskatchewan, Saskatoon, SK S7N 2Z4, Canada; 4 Prairie Diagnostic Services Inc., Saskatoon, SK S7N 5B4, Canada; yanyun.huang@usask.ca

**Keywords:** influenza A virus, H5N1, H7N9, pathogenesis in mice

## Abstract

Highly pathogenic avian influenza (HPAI) H5N1 and low pathogenic avian influenza (LPAI) H7N9 viruses pose a severe threat to public health through zoonotic infection, causing severe respiratory disease in humans. While HPAI H5N1 human infections have typically been reported in Asian countries, avian H7N9 human infections have been reported mainly in China. However, Canada reported a case of fatal human infection by the HPAI H5N1 virus in 2014, and two cases of human illness associated with avian H7N9 virus infection in 2015. While the genomes of the causative viruses A/Alberta/01/2014 (H5N1) (AB14 (H5N1)) and A/British Columbia/1/2015 (H7N9) (BC15 (H7N9)) are reported, the isolates had not been evaluated for their pathogenicity in animal models. In this study, we characterized the pathogenicity of AB14 (H5N1) and BC15 (H7N9) and found that both strain isolates are highly lethal in mice. AB14 (H5N1) caused systemic viral infection and erratic proinflammatory cytokine gene expression in different organs. In contrast, BC15 (H7N9) replicated efficiently only in the respiratory tract, and was a potent inducer for proinflammatory cytokine genes in the lungs. Our study provides experimental evidence to complement the specific human case reports and animal models for evaluating vaccine and antiviral candidates against potential influenza pandemics.

## 1. Introduction

Influenza A virus (IAV) is a segmented RNA virus that infects a wide variety of species including human, avian, swine, equine, and sea mammals. The segmented genome and wide host range enable IAV to undergo antigenic variations through gene reassortment, a mechanism called antigenic shift, which is responsible for the emergence of pandemic influenza viruses. The lack of proofreading by the viral RNA-dependent RNA polymerase frequently results in point mutations throughout the viral genome (especially in HA and NA genes), which can lead to antigenic drift, a causative mechanism of annual influenza epidemics [1,2]. Additionally, IAV also poses a severe threat to public health through zoonotic infection, because avian IAVs can directly cross the species barrier and infect humans, with avian H5N1 and H7N9 viruses being such examples. The first report of human infection by the highly pathogenic avian influenza (HPAI) H5N1 virus was in 1997 in Hong Kong; after a short period of disappearance, the virus re-emerged in 2003 in China [3]. Since then, sporadic human H5N1 viral infections have been reported in several countries, causing over 400 fatalities [4]. The low pathogenic avian influenza (LPAI) H7N9 virus circulated exclusively among poultry in China until 2013 when the first human infection was reported [5]. Similar to the HPAI H5N1 virus, human infection by the H7N9 virus is also associated with a severe and fatal respiratory disease. To date, the H7N9 virus has caused a total of six epidemic waves, having infected over 1600 humans with 623 fatalities [6].

In comparison to the human seasonal H1N1 and H3N2 viruses, the HPAI H5N1 virus induces more proinflammatory cytokines in the human alveolar and bronchial epithelial cells [7]. H5N1 viral infection also results in the early and excessive infiltration of macrophages and neutrophils in the lungs of infected mice [8]. Animal and human studies have shown that the combinatorial effect of the unrestrained high-level virus infection together with hypercytokinemia is attributable to the increased pathogenesis of H5N1 disease [9,10]. Although both H5N1 and H7N9 viral infections lead to fatality, the underlying mechanisms might be different [11]. An ex vivo study showed that the human H7N9 virus replicated efficiently in human bronchial epithelial cells, alveolar epithelial cells, and alveolar macrophages, with high titers similar to that of the H5N1 virus [12]. However, in animal studies, it has been reported that the H7N9 virus possesses a greater tropism for the respiratory epithelium than that of the H5N1 virus, and is not capable of inducing hypercytokinemia, which is characteristic of H5N1 viral infection [11]. Recently, a risk assessment of the fifth wave of H7N9 viral infection revealed that both the LPAI and HPAI H7N9 viruses were isolated from humans. Compared to the LPAI H7N9, the HPAI H7N9 virus was found to possess enhanced virulence, tropism for the brain tissue, as well as the capability to transmit by air droplets, concluding that the HPAI H7N9 virus has gained the ability to cause an H7N9 pandemic [13]. Thus, both H5N1 and H7N9 viruses are of significant pandemic concerns.

Both HPAI H5N1 and LPAI H7N9 human infections in North America were reported in Canada, in 2014 and 2015 respectively, shortly after the patients returned from China [14,15]. The A/Alberta/01/2014 (H5N1) (AB14 (H5N1)) strain isolate caused a fatal infection, whereas the A/British Columbia/1/2015 (H7N9) (BC15 (H7N9)) strain isolate caused an influenza illness from which the patients recovered. Phylogenetic analysis revealed mutations on the receptor binding site of the HA gene in AB14 (H5N1), which facilitated the direct jump from avian to humans [16]. However, for BC15 (H7N9), the analysis showed the genome to be similar to those of previous human H7N9 isolates, carrying clinically relevant markers on HA, PB2, and NA genes [15]. Although H5N1 and H7N9 viruses have been intensively studied, the virulence of these avian-origin influenza strains isolated from these Canadian travelers has been overlooked. The purpose of this study was to experimentally investigate the pathogenicity as well as the cytokine and chemokine gene transcription profile of the AB14 (H5N1) and BC15 (H7N9) strain isolates. We report that both AB14 (H5N1) and BC15 (H7N9) strains are highly lethal in mice. AB14 (H5N1) caused a systemic viral infection as well as an erratic proinflammatory cytokine and chemokine gene response in different organs. In contrast, the BC15 (H7N9) strain replicated efficiently only in the respiratory tract, and was a potent inducer for proinflammatory cytokines in the lungs. Overall, our study not only provides experimental evidence to complement the human case report, but also offers valuable animal models for evaluating vaccine and antiviral candidates against the potential H5 and H7 influenza pandemic viruses.

## 2. Materials and Methods

### 2.1. Cells and Viruses

Madin-Darby canine kidney (MDCK, ATCC, #CRL-2936) cells were maintained in minimal essential medium (MEM, Sigma-Aldrich, St. Louis, MO, USA) supplemented with 10% fetal bovine serum (FBS, Sigma-Aldrich) and gentamycin (50 µg/mL, Bio Basic, Markham, ON, Canada). MDCK cells were maintained at 37 °C in a humidified 5% CO_2_ incubator. Influenza A/Alberta/01/2014 (H5N1) (AB14 (H5N1)) and A/British Columbia/01/2015 (H7N9) (BC15(H7N9)) were kind gifts from Dr. Yan Li at the National Microbiology Laboratory, Public Health Agency of Canada. The viruses were propagated in MDCK cells and titrated by plaque assay. Propagated viruses were sequenced, and no mutations were found according to the reference sequences. All infectious experiments were conducted in Biosafety Containment Level 3 at the International Vaccine Centre at the University of Saskatchewan, Canada, under the guidelines of the Public Health Agency of Canada (PHAC) and the Canadian Food Inspection Agency (CFIA).

### 2.2. Ethics Statement

All animal procedures were approved by the Animal Care Committee (UACC) and Animal Research Ethics Board (AREB) of the University of Saskatchewan on 31 August 2017 (Animal Use Protocol #20170087) in accordance with the standards stipulated by the Canadian Council on Animal Care.

### 2.3. Mouse Experiments

For this study, 84 six-week-old female BALB/c mice (Charles River Laboratories, Saint-Constant, QC, Canada) were randomly divided into seven groups with 12 mice in each group. These groups were housed in separate cages in Biosafety Containment Level 3 seven days prior to infection. At seven weeks of age, each mouse was intranasally infected with 50 μL of 10^3^ PFU, 10^4^ PFU, and 10^5^ PFU of either the AB14 (H5N1) or BC15 (H7N9) strain isolates. A group of mice was mock infected with PBS. The mice were monitored daily for body weight, and on days 2 and 5 post-infection (d.p.i), three mice from each group were euthanized, to which the lung and spleen tissues were collected for viral titration as well as cytokine and chemokine profiling. Brain tissues were collected from euthanized mice on either 6 or 7 d.p.i. The rest of the mice were humanely euthanized when they dropped below 20% of their initial body weight.

### 2.4. Virus Isolation and Titration

Infectious lung, spleen, and brain tissues were processed immediately after collection as previously described [17]. Briefly, the tissues were homogenized in MEM supplemented with Penicillin-Streptomycin (Gibco, Thermo Fisher, ON, Canada) in the TissueLyser II (Qiagen, Hilden, Germany) at 25 Hz for 5 min, followed by centrifugation at 5000× *g* for 10 min at 4 °C. The supernatant was collected in screw-cap tubes and stored at −80 °C for further titration.

For virus titration by TCID_50_ assay, MDCK cells were plated in 96-well plates, and the supernatants of homogenized tissues were serially diluted in MEM and incubated with cells for 1 h. The inoculum was removed and supplemented with MEM containing 0.2% BSA and 1 µg/mL TPCK-trypsin (Sigma-Aldrich). The development of cytopathic effects (CPE) was observed and recorded every 24 h until 96 h post-infection. The TCID_50_ titer of each infectious sample was calculated by the Spearman–Kärber algorithm [18,19].

### 2.5. RNA Extraction and Quantitative RT-PCR (qRT-PCR)

Tissue samples of mice collected on 2 or 5 d.p.i. or upon necropsy were submerged into RNA later (Qiagen) and stored overnight at 4 °C. The following day, the tissue samples were transferred to screw-cap tubes containing one 5 mm stainless steel bead (Qiagen) and 1 mL of TRIzol Reagent (Invitrogen, Carlsbad, CA, USA). The samples were homogenized using the TissueLyser II (Qiagen) at 25 Hz for 5 min, followed by centrifugation at 5000× *g* for 10 min at 4 °C. The supernatant was then transferred to new tubes for RNA extraction by the TRIzol method (Invitrogen).

To determine mRNA levels of various cytokines and chemokines induced by AB14 (H5N1) and BC15 (H7N9) viral infection, qRT-PCR was performed on total RNA of the samples collected from mice infected with 10^3^ PFU of the respective virus, as previously described [20] with the following modifications. Briefly, a 500 µg portion of RNA was reverse transcribed with oligo (dT) and SuperScript III Transcriptase (Invitrogen) to obtain total mRNA. qPCR was performed on a StepOnePlus^TM^ Real-Time PCR system (Applied Biosystems, CA, USA) with the Power SYBR Green PCR Master Mix (Applied Biosystems). Cytokine mRNA levels were normalized to that of the housekeeping gene HPRT and expressed using the ∆ΔCT method relative to the PBS group. All sequences of qPCR primers are listed in Table 1.

### 2.6. Histopathology

The left side of the lung was fixed with 10% natural buffered formalin, processed for hematoxylin and eosin (H&E) staining, and assessed in a blind manner by a board-certified veterinary pathologist as previously described [21].

## 3. Results

### 3.1. Survival Rate and Body Weight Loss of Mice Infected with the HPAI H5N1 and LPAI H7N9 Strain Isolates

**Figure 1:**
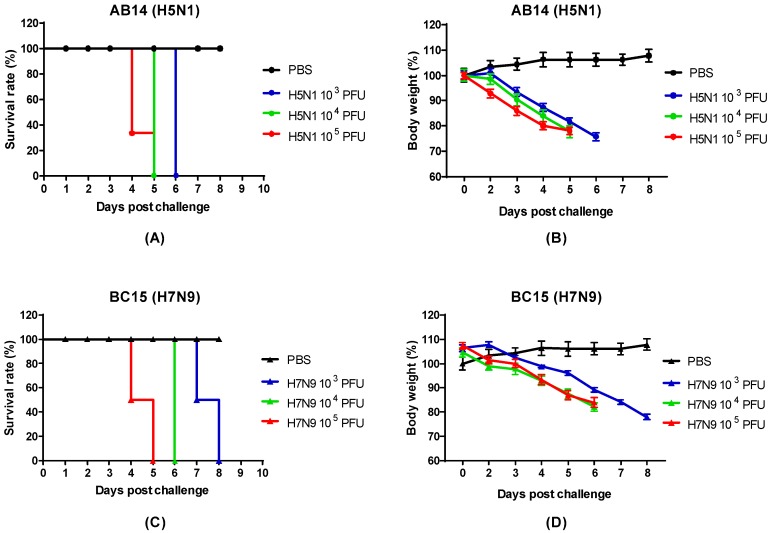
Survival rate and body weight loss for the AB14 (H5N1) and BC15 (H7N9) strain isolates. The survival rates for (**A**) AB14 (H5N1) and (**C**) BC15 (H7N9) as well as body weight changes for (**B**) AB14 (H5N1) and (**D**) BC15 (H7N9) were determined in BALB/c mice (*n* = 6 per group) infected with 10^3^ PFU, 10^4^ PFU, and 10^5^ PFU of the two different strain isolates.

To determine the pathogenicity of the AB14 (H5N1) and BC15 (H7N9), BALB/c mice were intranasally inoculated with PBS or either AB14 (H5N1) or BC15 (H7N9) at three different doses (10^3^ PFU, 10^4^ PFU, and 10^5^ PFU). Survival and body weight loss were monitored for 10 days (Figure 1). Mice infected with PBS survived the duration of the trial and gained weight as the days progressed. In contrast, mice infected with the different doses of either AB14 (H5N1) or BC15 (H7N9) showed rapid body weight loss and severe mortality rates. Mice infected with 10^4^ PFU or 10^5^ PFU of AB14 (H5N1) exhibited rapid weight loss greater than 20% of their initial body weight within four days post-infection (d.p.i.). Furthermore, over 60% of mice infected with 10^5^ PFU of AB14 (H5N1) had succumbed by 4 d.p.i. (Figure 1A,B). By 5 d.p.i., 100% of mice infected with 10^4^ PFU and 10^5^ PFU were humanely euthanized due to severe body weight loss. Albeit mice infected with 10^3^ PFU displayed the slowest rate of body weight loss, they all reached a humane endpoint by 6 d.p.i. With regard to BC15 (H7N9), mice infected with 10^3^ PFU displayed the slowest decline of body weight, losing over 20% of their initial body weight by 8 d.p.i. Furthermore, 50% of mice infected with 10^3^ PFU reached a humane endpoint by 7 d.p.i., while the other 50% reached this endpoint by 8 d.p.i. (Figure 1C,D). In contrast, mice infected with 10^4^ PFU and 10^5^ PFU had steeper body weight losses and higher mortality rates. In the 10^4^ PFU group, 100% of mice reached a humane endpoint by 6 d.p.i., while in the 10^5^ PFU group, 50% of the mice by 4 d.p.i. and the other 50% by 5 d.p.i. reached this endpoint. These results demonstrate that both the AB14 (H5N1) and BC15 (H7N9) strain isolates are highly virulent in mice without the need of prior adaptation even at the lowest dose of 10^3^ PFU.

### 3.2. Histopathology of the Mouse Lung

**Figure 2:**
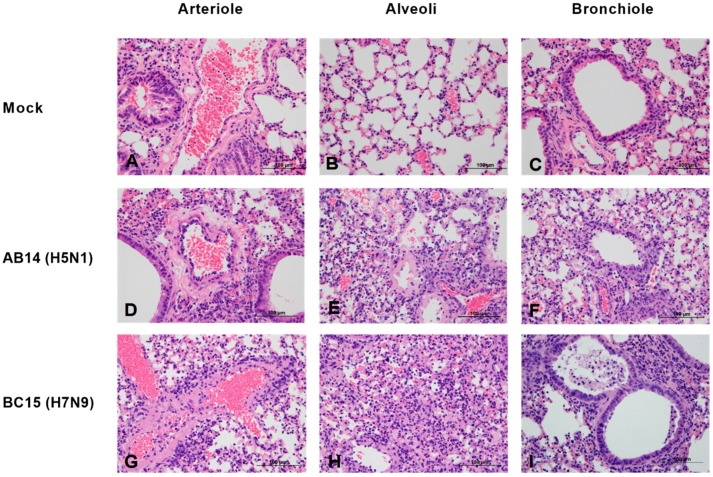
Lung histopathology of mice after infection with AB14 (H5N1) and BC15 (H7N9) strain isolates. Lung samples were fixed, sectioned, and stained with hematoxylin and eosin. (**A**–**C**) Tissues from mock-infected lungs. (**D**–**F**) Tissues from mice infected with AB14 (H5N1) showing infiltration of inflammatory cells into the (**D**) wall of the arteriole, (**E**) alveolar walls, and (**F**) bronchiolar epithelium affected by necrosis. (**G**–**I**) Tissues from mice infected with BC15 (H7N9) showing necrotic debris and inflammatory cells in the (**G**) wall of the arteriole, (**H**) collapsed alveoli, as well as degeneration and necrosis of the (**I**) bronchiolar epithelium. Scale bar represents 100 µm.

To examine the levels of pulmonary pathology from AB14 (H5N1) and BC15 (H7N9) infection, we performed a histopathology study on the lungs of mice infected with 10^3^ PFU that reached a humane endpoint on 6 or 7 d.p.i. Mice infected with both AB14 (H5N1) and BC15 (H7N9) strain isolates showed bronchointerstitial pneumonia, with vasculitis (Figure 2). Specifically, the walls of the arterioles in infected mice were found to be infiltrated with inflammatory cells, and contained some necrotic debris (Figure 2, Panels D and G). In addition, viral infection also led to moderate damage to the mice alveoli and bronchiolar. In the alveoli of infected mice, we found moderate thickening of the alveolar walls due to congestion as well as some inflammatory infiltrate (Figure 2, Panels E and H). The alveolar space was filled with edema, and contained small to moderate numbers of mixed neutrophils and macrophages. Occasionally, hyaline membranes lining the alveoli were observed (Figure 2, Panel E). Multifocally, the bronchiolar epithelium was necrotic, and the lumen was filled with some necrotic debris and inflammatory cells as seen in the alveolar space (Figure 2, Panels F and I).

### 3.3. Replication Efficiency of the HPAI H5N1 and LPAI H7N9 Strain Isolates in Different Organs of Mice

**Figure 3:**
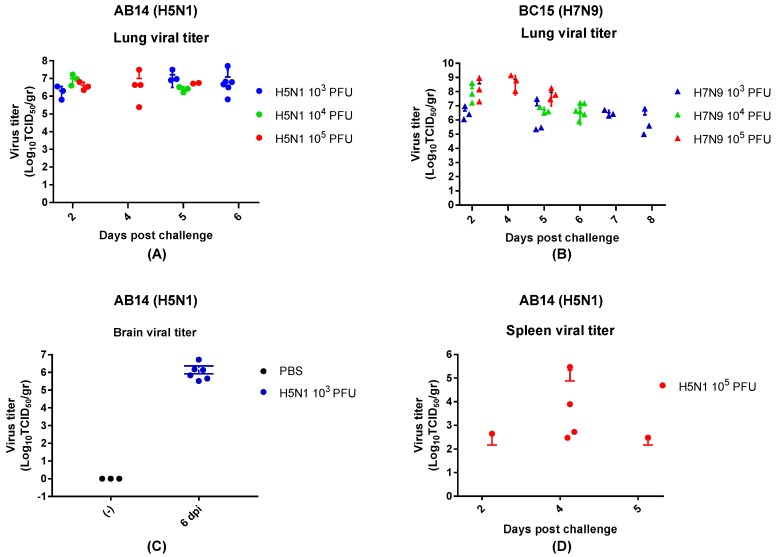
Viral titration of the mouse lung, spleen, and brain for the AB14 (H5N1) and BC15 (H7N9) strain isolates. Mice were intranasally infected with 10^3^ PFU, 10^4^ PFU, or 10^5^ PFU of AB14 (H5N1) or BC15 (H7N9). The lung, spleen, and brain tissues were collected and homogenized for virus titration by TCID_50_ assay. Lung viral titration from (**A**) AB14 (H5N1) and (**B**) BC15 (H7N9) infection for all doses. Brain viral titration from (**C**) AB14 infection (H5N1). Spleen viral titration from (**D**) AB14 (H5N1) infection. Please note that BC15 (H7N9) was not detected in the spleen and brain of mice infected by all three doses.

To investigate the replication efficiency and tissue tropism of both strain isolates, mouse lung, spleen, and brain tissues were collected at predetermined days, as well as when the mice reached a critical endpoint (Figure 3). In all groups of mice infected with AB14 (H5N1), the peak lung viral titers were reached by 2 d.p.i., which remained at high levels throughout the trial. The mean titers on 5 d.p.i. were 10^7.2^, 10^6.4^, and 10^6.7^ TCID_50_/g for 10^3^ PFU, 10^4^ PFU, and 10^5^ PFU, respectively (Figure 3A). Brain samples taken from the lowest dose group (10^3^ PFU) had very high viral titers on 6 d.p.i., with a mean virus titer of 10^7^ TCID_50_/g (Figure 3C). Note that the brain samples were only harvested at the endpoint. Spleen viral titers remained at an approximate level of 10^2.5^ TCID_50_/g from 2 d.p.i. until 5 d.p.i. (day 2: 10^2.64^ TCID_50_/g, day 5: 10^2.46^ TCID_50_/g) in the mice infected with 10^5^ PFU of the AB14 (H5N1) strain isolate (Figure 3D). Interestingly, spleen viral titers were not detected for the two lower dose groups (10^3^ PFU and 10^4^ PFU). 

In BC15 (H7N9) viral infected lung, the 10^3^ PFU group reached a titer of approximately 10^6.5^ TCID_50_/g by 2 d.p.i., and remained at this titer throughout the trial. The 10^4^ PFU group displayed a similar trend, with high titers present at 2 d.p.i. (10^8^ TCID_50_/g), which decreased by 5 and 6 d.p.i. (10^6.5^ TCID_50_/g). The 10^5^ PFU group had the highest titer at 2 d.p.i. (10^8^ TCID_50_/g), which remained at this level throughout the trial (Figure 3B). The BC15 (H7N9) strain isolate was not detected in the spleen and brain of mice infected by all three doses by TCID_50_ assay.

### 3.4. Cytokine and Chemokine Profiling in the Mouse Lung

**Figure 4:**
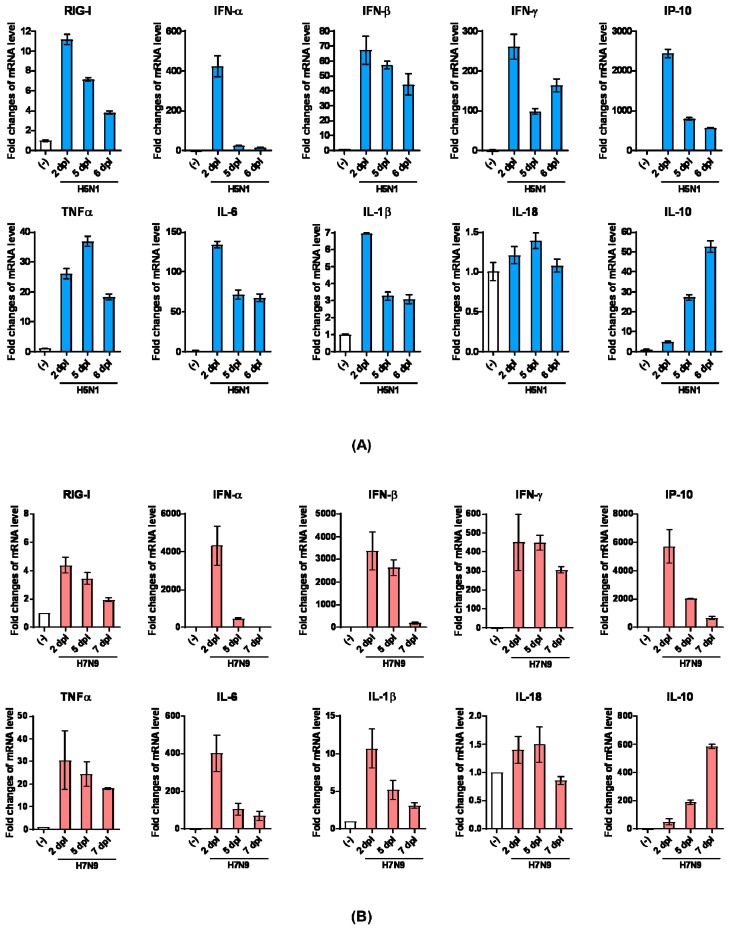
Innate immune receptor RIG-I, as well as cytokine and chemokine gene transcription levels in the lungs of mice infected with the AB14 (H5N1) and BC15 (H7N9) strain isolates. Cytokine and chemokine mRNA levels of RIG-I, IFN-α, IFN-β, IFN-γ, IP-10, TNF-α, IL-6, IL-1β, IL-18, and IL-10 from virus-infected lungs with (**A**) AB14 (H5N1) and (**B**) BC15 (H7N9) (*n* = 3 mice per virus group) were measured by qRT-PCR. Samples were harvested on the indicated d.p.i.; each sample was tested in triplicate.

To understand the pathogenesis and immune response to AB14 (H5N1) and BC15 (H7N9) viral infection, we assessed the innate immune receptor RIG-I, as well as the cytokine and chemokine gene expression in the lungs and brains of mice infected with the lowest dose (10^3^ PFU) of both strain isolates by qRT-PCR. To start, we investigated a group of genes that encode interferons as well as the innate immune sensor RIG-I, a major pattern recognition receptor that recognizes influenza viral infection and activates the interferon response [22,23]. IFN-α and IFN-β are both type I interferons which play essential roles in antiviral defense [24]. IFN-γ is a type II interferon that is the first cytokine produced in response to foreign invaders, and is an important activator of macrophages and natural killer (NK) cells. IFN-γ also has antiviral activity, and inhibits the proliferation of Th2 cytokines (IL-4, IL-5, and IL-6) [25]. In the AB14 (H5N1) infected lungs (Figure 4A), gene expression of RIG-I peaked on 2 d.p.i., which then decreased by 5 and 7 d.p.i. IFN-α gene expression was drastically upregulated on 2 d.p.i., and then sharply dropped on 5 and 7 d.p.i. IFN-β mRNA levels increased over 60-fold on 2 d.p.i., which then slightly decreased but remained at moderately high levels on 5 and 7 d.p.i. The type I IFN gene transcription levels showed similar upregulating patterns in concordance with that of RIG-I. IFN-γ had the highest upregulation by 2 d.p.i., which dropped moderately by 5 d.p.i., and then increased slightly by 7 d.p.i. The transcription of the gene encoding interferon-gamma-inducing protein 10 (IP-10), a cytokine produced in response to IFN-γ, was significantly upregulated (over 2000-fold) on 2 d.p.i., and then remained at relatively high levels, consistent with the IFN-γ mRNA upregulation pattern. Next, we profiled the gene transcription of proinflammatory (TNFα, IL-6, IL-1β, and IL-18) and anti-inflammatory (IL-10) cytokines. TNFα is a proinflammatory cytokine produced in response to the foreign invasion, and is often the main cytokine produced by macrophages following infection [26]. IL-6 and IL-1β are the main contributors to severe lung inflammation in both humans and poultry infected by influenza virus [27]. Both TNFα and IL-6 mRNA were markedly upregulated upon AB14 (H5N1) infection and remained at these elevated levels. IL-1β mRNA was upregulated 7-fold on 2 d.p.i., which then returned to slightly higher levels than that of the uninfected control. IL-18 mRNA remained at mock levels upon infection. On the contrary, the mRNA level of the anti-inflammatory cytokine IL-10 was gradually upregulated after infection, and peaked on 7 d.p.i.

In the lungs of BC15 (H7N9) infected mice, substantially upregulated mRNA levels of interferon genes (IFN-α, IFN-β, and IFN-γ), interferon responsive cytokine gene (IP-10), and proinflammatory cytokine genes (TNFα and IL-6) were obtained on 2 d.p.i. compared to the uninfected control (Figure 4B). IFN-α and IFN-β mRNA levels substantially dropped by 7 d.p.i., whereas mRNA levels of RIG-I, IFN-γ, and TNFα remained relatively unchanged throughout the duration of the trial. Similar to what we observed for AB14 (H5N1), the IL-18 gene transcript was not upregulated in response to viral infection, whereas the gene transcription of the anti-inflammatory cytokine IL-10 dramatically increased from 2 to 7 d.p.i.

**Figure 5:**
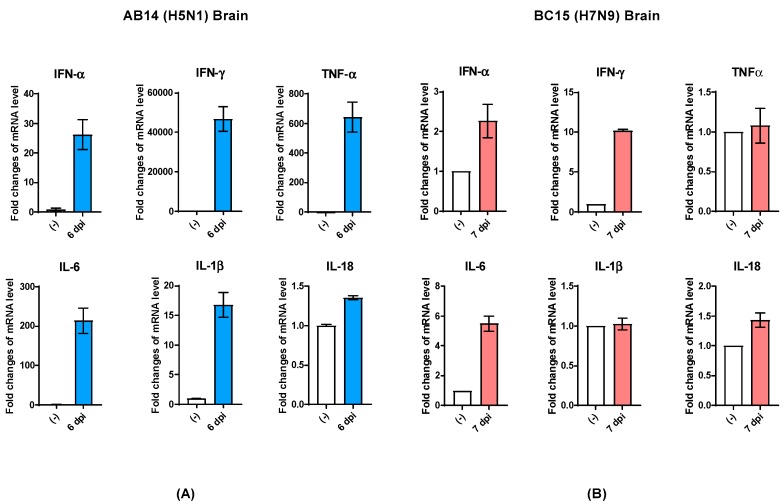
Cytokine gene transcription profile in the brains of mice infected with the AB14 (H5N1) and BC15 (H7N9) strain isolates. Cytokine transcription levels of IFN-α, IFN-γ, TNF-α, IL-6, IL-1β, and IL-18 from virus-infected brains with (**A**) AB14 (H5N1) and (**B**) BC15 (H7N9) (*n* = 3 mice per virus group). Samples were harvested on the indicated d.p.i; each sample was tested in triplicate.

Besides cytokine and chemokine gene transcription analysis in the lungs of viral infected mice, we also analyzed select cytokine transcription levels in the brains of mice harvested at the endpoint. In the brains of mice infected with AB14 (H5N1), the transcription of IFN genes (IFN-α and IFN-γ) as well as proinflammatory cytokine genes (TNFα, IL-6, and IL-1β) was dramatically upregulated compared to the uninfected control (Figure 5A). Among all genes activated in the brain tissue upon infection, IFN-γ and TNFα were the most highly transcribed cytokines associated with HPAI AB14 (H5N1) infection. Similar to the cytokine regulation observed in the mouse lung, IL-18 transcripts did not change upon AB14 (H5N1) infection when compared to the uninfected control (Figure 5A). In contrast, the brains of mice infected with BC15 (H7N9) had only moderate upregulation of IFN-α, IFN-γ, and IL-6 gene transcription, which was marginal compared to the degree of upregulation observed in the AB14 (H5N1) viral infected brain. The transcripts of the other inflammatory cytokine genes, TNFα, IL-1β, and IL-18, did not change when compared to the uninfected control (Figure 5B).

## 4. Discussion and Conclusions

Following the reporting of the human HPAI (H5N1) and LPAI (H7N9) infection in Canada, the characterization on the causative strain isolates associated with these human infection cases, AB14 (H5N1) and BC15 (H7N9), was reported. Pabbaraju et al. analyzed the full genome of AB14 (H5N1) and assessed its molecular markers of pandemic risk [16]. Skowronski et al. conducted the serological and phylogenetic analysis of BC15 (H7N9), reporting that this virus belongs to the clade W2-C, which clusters with both the 2014–2015 H7N9 human isolates from the Jiangsu, Zhejiang, and Fujian Provinces of China as well as the 2014 chicken isolated from Jiangsu Province [15]. Despite the analysis on these two Canadian isolates, the infectious and immunological properties as well as the histopathological correlation to disease remained elusive. In this study, we aimed to fill this knowledge gap, and to provide insightful information on the pathogenesis of the AB14 (H5N1) and BC15 (H7N9) strain isolates in mice.

To ensure that the AB14 (H5N1) and BC15 (H7N9) strain isolates would induce disease in mice, we chose to infect them with three different doses (10^3^ PFU, 10^4^ PFU, and 10^5^ PFU). We found that even at the lowest dose (10^3^ PFU), both isolates were lethal to mice. AB14 (H5N1) caused all mice to reach a humane endpoint by 6 d.p.i., whereas BC15 (H7N9) infected mice survived one to two days longer than the mice infected with AB14 (H5N1). This observation is in contrast to the various seasonal or the 2009 pandemic influenza viruses [28,29]. Groves et al. reported that mice infected with 5 × 10^3^ PFU of influenza A/England/195/2009 (H1N1) showed mild weight loss over 7 days, whereas mice infected with 10^3^ PFU of influenza A/England/691/2010 (H3N2) did not show any signs of disease [28]. In the study reported by Rowe et al., infection of BALB/c mice with 10^4^ EID_50_ of the 2009 pandemic virus did not cause any death when using 20% total body weight loss as an endpoint [29]. Furthermore, we could detect the AB14 (H5N1) strain in multiple organs, including the lung, spleen, and brain, with consistently high titers in the lung and brain tissues. However, we could only detect the BC15 (H7N9) strain in the mouse lung, with no detectable virus in the spleen and brain by TCID_50_ assay. These results are in agreement with the previous reports that while H5N1 virus has the ability to replicate systemically and spread efficiently to non-respiratory tissues, H7N9 virus replicates well in the upper respiratory tract, covering the nasal passages, nasopharynx-associated lymphoid tissues [11], human bronchus, and the lungs [12]. Research has shown that avian IAV strains need to acquire adaptation mutations in order to be infectious in mammals, with one such adaptation being in the polymerase, PB2. This adaptation resides at the amino acid at position 627 of PB2, and determines the viral replication efficiency in different hosts. It is well characterized that avian strains carry a glutamic acid (E) at this position referred to as 627E, whereas mammalian strains carry a lysine (K), namely, 627K [30]. The BC15 (H7N9) strain isolate consists of PB2 encoding 627K, which may explain its ability to replicate well in the human respiratory tract. In contrast, the AB14 (H5N1) strain isolate consists of PB2 possessing the avian signature, namely, 627E. However, our data have shown that while AB14 (H5N1) replicates efficiently without any adaptation in mice, it replicates less efficiently in tissue culture (Lu and Zhou unpublished data). In addition to the PB2 protein, HA protein is also a key viral factor that determines whether an avian virus can replicate well in mammals. It is reported that AB14 (H5N1) HA contains two novel mutations, R189K and G221R, which are located in the immediate receptor-binding pocket [16]. It was speculated that these mutations arose in an avian H5N1-infected patient, which was also associated with severe illness and spread of the virus to the brain. We observed that the AB14 (H5N1) strain isolate replicated efficiently in the mouse brain, which will provide a model for further investigation of the functional role of G221R in avian influenza HA contributing to viral pathogenesis in mammals.

Research has shown that the dysregulation of the innate immune responses that results in an unusual proinflammatory cytokine production often contributes to the pathogenicity of both the HPAI H5N1 virus and the 1918 Spanish Flu pandemic virus [8,31]. Our data show that although at different magnitudes, infection by both AB14 (H5N1) and BC15 (H7N9) induced dramatically elevated levels of interferon genes and proinflammatory cytokine gene expression in the lungs. These levels peaked on 2 d.p.i., and then decreased but remained at moderate levels as the infection progressed. Concomitantly, both strain isolates induced increased gene expression of the anti-inflammatory cytokine IL-10, which peaked on 6 and 7 d.p.i., for both the AB14 (H5N1) and BC15 (H7N9) strain isolates, respectively. There has been evidence showing IL-10 to inhibit several proinflammatory cytokines and chemokines [32], and that it may therefore actually hinder clearance of the virus [33]. When comparing the transcription levels of various cytokines, it was found that most cytokine genes were sharply upregulated in the tens- to hundreds-fold. Interestingly, the two inflammatory cytokines IL-1β and IL-18, whose maturation is regulated by inflammasome activity [34], were not significantly upregulated at the mRNA level; IL-1β mRNA was only moderately upregulated, whereas IL-18 mRNA was not upregulated at all. This finding thus warrants further investigation into the mature IL-1β and IL-18 protein levels, in order to understand their roles in mediating avian IAV-induced pathogenesis. Interestingly, it has been reported that in comparison to other H5N1 strain isolates that are associated with severe human respiratory disease, H7N9 strains isolated from human infections from 2013 to 2015 are poor proinflammatory cytokine and chemokines inducers in mammalian models [35]. However, our results showed that BC15 (H7N9) is a potent inducer of proinflammatory cytokines in the mouse lung. This finding is consistent with the study of human patients infected with the H7N9 virus, where elevated cytokine and chemokine production was observed [36]. With regard to the cytokine gene expression in the mouse brain, we found that AB14 (H5N1) dramatically upregulated IFN-α, IFN-γ, TNF-α, and IL-6 transcription in the brains of mice that reached a humane endpoint. This finding possibly indicates cytokine hyperinduction in the brains of mice infected with AB14 (H5N1), which may contribute to the pathogenesis. In contrast, only moderate upregulation of both IFN-γ and IL-6 transcripts was observed in the brains of mice infected with BC15 (H7N9). This finding reflects the viral titers found in the infected brains of the mice, such that while mice infected with AB14 (H5N1) had high titers, mice infected with BC15 (H7N9) had no detectable virus in the brain. 

Taken together, we showed that the AB14 (H5N1) strain isolate replicates efficiently without prior adaptation not only in the mouse lung, but also in other non-respiratory tissues such as the spleen and brain. The high viral load and hypercytokinemia in multiple organs contributed to the severity of the disease associated with AB14 (H5N1) infection. Similarly, the BC15 (H7N9) strain is also highly pathogenic in mice, but its replication seemed to be more constrained to the respiratory tract. The severity of both AB14 (H5N1) and BC15 (H7N9) disease as well as the significant lung pathology observed may be a result of the unusual upregulation of proinflammatory cytokines including TNF-α, IP-10, and IL-6. Our findings not only contribute to a better understanding of the pathogenesis of the strain isolates associated with Canadian human cases of avian H5N1 and H7N9 virus infection, but also provide animal models for testing vaccine and antiviral candidates for viruses that are of significant public health concerns.

## Figures and Tables

**Table 1:** List of primers used in RT-qPCR studies in mice. All primers have been validated to have greater than 95% of amplification efficiency. The expression levels of cytokine mRNA were normalized to the expression of the housekeeping gene HPRT. F: forward primer; R: reverse primer.

Name	Sequence (5′-3′)
IFN-γ-F	TCAAGTGGCATAGATGTGGAAGAA
IFN-γ-R	TGGCTCTGCAGGATTTTCATG
IFN-α-F	CCTGTGTGATGCAACAGGTC
IFN-α-R	TCACTCCTCCTTGCTCAATC
IFN-β-F	ATCATGAACAACAGGTGGATCCTCC
IFN-β-R	TTCAAGTGGAGAGCAGTTGAG
IP-10-F	ATGACGGGCCAGTGAGAATG
IP-10-R	GAGGCTCTCTGCTGTCCATC
TNFα-F	AGGCACTCCCCCAAAAGATG
TNFα-R	CTGCCACAAGCAGGAATGAG
IL-1β-F	GTGTGGATCCCAAGCAATAC
IL-1β-R	GTCCTGACCACTGTTGTTTC
IL-18-F	TGGTTCCATGCTTTCTGGACTCCT
IL-18-R	TTCCTGGGCCAAGAGGAAGTGATT
IL-6-F	GTGGCTAAGGACCAAGACCA
IL-6-R	TAACGCACTAGGTTTGCCGA
IL-10-F	GCTGCCTGCTCTTACTGACT
IL-10-R	CTGGGAAGTGGGTGCAGTTA
RIG-I-F	CCTCCCATCTCCTTCATGACA
RIG-I-R	CCACCTACATCCTCAGCTACATGA
HPRT-F	GATTAGCGATGATGAACCAGGTT
HPRT-R	CCTCCCATCTCCTTCATGACA
